# High spectral resolution of gamma-rays at room temperature by perovskite CsPbBr_3_ single crystals

**DOI:** 10.1038/s41467-018-04073-3

**Published:** 2018-04-23

**Authors:** Yihui He, Liviu Matei, Hee Joon Jung, Kyle M. McCall, Michelle Chen, Constantinos C. Stoumpos, Zhifu Liu, John A. Peters, Duck Young Chung, Bruce W. Wessels, Michael R. Wasielewski, Vinayak P. Dravid, Arnold Burger, Mercouri G. Kanatzidis

**Affiliations:** 1 Department of Chemistry, Northwestern University, Evanston, IL 60208 USA; 2 Department of Life and Physical Sciences, Fisk University, Nashville, TN 37208 USA; 3 Department of Materials Science and Engineering, Northwestern University, Evanston, IL 60208 USA; 4 Department of Chemistry and Physics, Chicago State University, Chicago, IL 60628 USA; 5 Materials Science Division, Argonne National Laboratory, Argonne, IL 60439 USA

## Abstract

Gamma-ray detection and spectroscopy is the quantitative determination of their energy spectra, and is of critical value and critically important in diverse technological and scientific fields. Here we report an improved melt growth method for cesium lead bromide and a special detector design with asymmetrical metal electrode configuration that leads to a high performance at room temperature. As-grown centimeter-sized crystals possess extremely low impurity levels (below 10 p.p.m. for total 69 elements) and detectors achieve 3.9% energy resolution for 122 keV ^57^Co gamma-ray and 3.8% for 662 keV ^137^Cs gamma-ray. Cesium lead bromide is unique among all gamma-ray detection materials in that its hole transport properties are responsible for the high performance. The superior mobility-lifetime product for holes (1.34 × 10^−3^ cm^2^ V^−1^) derives mainly from the record long hole carrier lifetime (over 25 μs). The easily scalable crystal growth and high-energy resolution, highlight cesium lead bromide as an exceptional next generation material for room temperature radiation detection.

## Abstract (web summary)

Detection and spectroscopic measurements of gamma-ray used to rely on expensive materials such as CdZnTe crystals. Here He et al. develop a melt method to grow large size CsPbBr_3_ perovskite crystals and the devices achieve low cost, high energy resolving capabilities and stability.

## Introduction

High performance, low cost nuclear radiation detectors based on compound semiconductors are a highly sought technology over many decades because of their broad potential applications in the fields of homeland security (nonproliferation of nuclear materials), industrial and medical imaging, and fundamental scientific research^1–3^. For such applications, gamma rays (γ-rays) must be detected with high-spectroscopic resolution to discriminate the characteristic γ-ray spectrum used to identify radionuclides contained in the source. There are two main detection modes for γ-ray radiation, optical-based scintillators and electronic-based semiconductors. Compared to scintillation detectors, which suffer from the intrinsic nonproportionality of the light yield making it challenging to measure accurate γ-ray energies, the output pulse height from on a semiconductor detector is just proportional to the charge that is induced over a specific time interval and thus has superior accuracy in energy resolution^4,5^. Therefore, the quest for semiconductor materials for low cost effective radiation detectors has existed from the dawn of the semiconductor era and persists to the present date^6^.

Prospective semiconductors need to fulfill simultaneously several desirable properties. The detector material should be: capable of interacting strongly with the high-energy photons; operable under high electric field with negligible leakage current; allowing for the photo-generated carriers to drift through the detector volume unimpeded, this means they must have extremely low concentration of charge trapping and electrically active defects; and must be easily upscalable to low cost^7^. On this premise, only few inorganic compounds, specifically Cd_1−*x*_Zn_*x*_Te (CdZnTe), CdTe, TlBr, and α-HgI_2_, are able to combine some of the above-mentioned characteristics and demonstrate a spectroscopic response^1,8–11^. However, each suffers from persistent unsolved issues associated with difficulties encountered in the crystal growth or in device operation^1,12,13^. For instance, secondary phases are always present in detector-grade CdZnTe crystals, such as inclusions and precipitates, and can degrade the local charge transport properties and the uniform response of detectors^14,15^. From the above compounds only CdZnTe, which has been in continuous development since the 1970s, has been commercialized for room temperature applications, albeit it at a high cost that limits widespread use.

Since 2012, halide perovskites with a general formula of AMX_3_, where A^+^ = Cs, CH_3_NH_3_, HC(NH_2_)_2_, M^2+^ = Ge, Sn, Pb, and X^−^ = Cl, Br, I, have attracted intense interest because of facile synthesis and processing, as well as impressive optical and electronic properties, such as an extremely low density of defects, excellent carrier transport, and surprisingly high defect tolerance^16–21^. However, in general, organic–inorganic hybrid perovskites intrinsically have structural disorder in the strongly polar organic cations and structurally dynamic inorganic frameworks, causing long-term chemical instability^18,20,22^.

The all-inorganic perovskite CsPbBr_3_ features a favorable electronic structure with a large bandgap of 2.3 eV, broad valence and conduction bands and can grow in large crystal sizes from its melt, as well as from solution. It also offers far greater long-term stability because it lacks organic molecules^23–27^. Furthermore, CsPbBr_3_ is a high *Z* compound with effective atomic number *Z*_eff_ of 65.9, which is much higher than CdZnTe (50.2). Thus, the attenuation coefficients of CsPbBr_3_ for γ-rays are higher than CdZnTe, leading to even higher stopping power especially in the high energy region (100–500 keV, Supplementary Figure 1). We initiated our research on CsPbBr_3_ in 2013 as a potential hard radiation detector material^23^. However, our previous and subsequent works did not yet deliver the detection of highly resolved γ-rays^24–26^.

Here we report a modified melt growth method for CsPbBr_3_, which results in very high purity material capable of detecting γ-rays as we demonstrate with ^57^Co 122 keV and ^137^Cs 662 keV γ-rays (as well as ^22^Na and ^241^Am γ-rays). In addition to achieving high crystal quality of CsPbBr_3_, we accomplish this by applying a special device architecture that dramatically reduces the leakage current under large electric field. We have thus produced CsPbBr_3_ planar detectors reproducibly, which show the best spectral resolution (3.8–3.9%) obtained for any halide perovskite and non-perovskite material to date and comparable to commercial samples of the benchmark CdZnTe planar detector which gives 4.1% for with ^57^Co 122 keV but can hardly resolve the 662 keV ^137^Cs γ-rays^28^.

## Results

### Improved single crystal growth

**Fig. 1:**
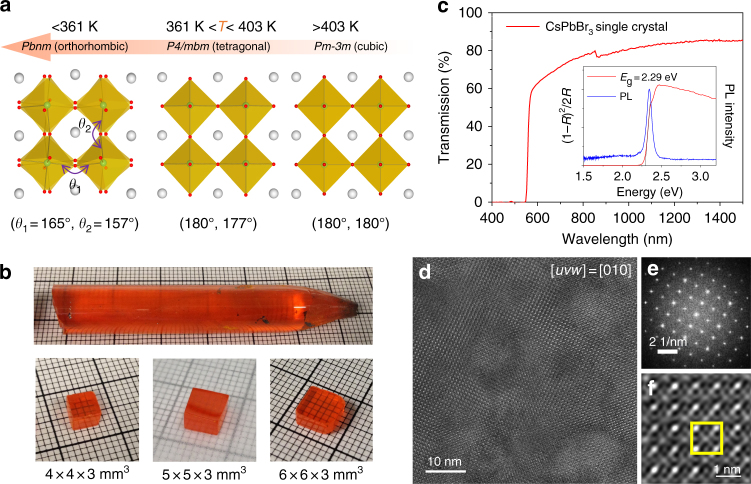
Crystal growth and general properties of CsPbBr_3_. **a** Nondestructive phase transitions in CsPbBr_3_ during cooling involving small rotations and tilts of PbBr_6_ octahedral (associated with angles *θ*_1_ and *θ*_2_). The Cs, Pb, and Br atoms are depicted as gray, green, and red spheres, respectively. **b** As-grown single crystal ingot with a diameter of 11 mm, and the single crystal wafers with different sizes, 4 × 4 × 3 mm^3^, 5 × 5 × 3 mm^3^, and 6 × 6 × 3 mm^3^. The smallest grid size corresponds to 1 mm. The pure orthorhombic phase of the crystal was confirmed by powder X-ray diffraction in Supplementary Figure 2. **c** Optical transmission spectrum for CsPbBr_3_ single crystals with size of 5 × 5 × 3 mm^3^. Insets are the optical absorption spectrum obtained from diffuse reflectance measurement on ground CsPbBr_3_ crystals and the steady-state PL of CsPbBr_3_ single crystal excited at 440 nm. **d** High resolution TEM (HRTEM) with selected area electron diffraction (SAD) in **e**, and magnified lattice image in **f**. Yellow squares in **f** are drawn to compare HRTEM image and simulations in Supplementary Figure 3

CsPbBr_3_ exhibits two nondestructive phase transitions at relatively low temperatures. The first phase transition which occurs around 130 °C from cubic (*Pm-3m*) to tetragonal (*P4/mbm*) is a first-order phase transition. This is followed by a second order transition around 88 °C to the orthorhombic (*Pbnm*) phase, which is stable at room temperature, Fig. 1a ^23^. Such phase transitions can induce residual stress or mechanical deformations and can lead to the formation of cracks or sub-grain boundaries or twins. Those structural defects could be detrimental to detector performance, and thus should be mitigated as much as possible during crystal growth.

One of the dramatic improvements achieved in this work is the optimization of our crystal growth process that can produce large crack-free single crystal ingots with diameter of 11 mm and length over 6 cm (Fig. 1b). We stress that the detailed kinetic parameters during the crystal growth process are extremely important. The melt temperature, temperature gradient, and cooling strategies are critical and have been optimized over multiple growth runs. The single crystal ingot in Fig. 1b was grown with a slow translation velocity of 0.5 mm h^−1^ with the specific overheating temperature and temperature gradient of 15 K and 9 K cm^−1^, respectively. The cooling strategy is particularly important for avoiding structural defects due to the low temperature phase transitions. The translation speed after crystallization was to keep the cooling rate very low at about 5 K h^−1^ and produced an orange-red transparent single crystal ingot free of visible cracks or grain boundaries. Supplementary Figure 4 illustrates the sensitivity of single crystal CsPbBr_3_ to the growth parameters by comparing the transmission spectra and detector performance of crystals grown with suboptimal growth speeds and cooling rates. The ingot was then cut and fabricated into wafers with regular shapes for detectors.

Compared to solution growth in organic solvents^25^, crystal growth from melt has significant advantages, such as control and amenability to scale-up. Moreover, melt growth under vacuum conditions inhibits the unintended contamination from solutions leading to higher purity level and better crystalline quality. The impurity levels in our CsPbBr_3_ single crystals, for up to 69 elements, were analyzed by the glow discharge mass spectrometry technique and their concentrations are listed in Supplementary Table 1. The total-impurity concentration for all elements is below 10 p.p.m. The impurities with concentration above 0.5 p.p.m. are Rb (1.3 p.p.m.), Ca (0.72 p.p.m.), Cl (6.8 p.p.m.), and I (less than 0.8 p.p.m.), which consist of over 90% of the total-impurity concentration. Those impurities are highly miscible with CsPbBr_3_ and likely incorporated from the precursor materials. These results confirm that the CsPbBr_3_ single crystal is highly chemically pure.

The optical transmittance for a 3 mm thick crystal exceeded 65% when the wavelength was over 600 nm and exceeded 80% when over 1000 nm (Fig. 1c). The high transmittance indicates that the as-grown single crystals do not have any significant concentration of optical absorption or scattering centers below the bandgap energy of 2.29 eV. The steady-state photoluminescence (PL) for our CsPbBr_3_ single crystal corresponds well to the previously observed bound exciton emission^29^ at 2.34 eV, while the time-resolved photoluminescence (TRPL) indicated biexponential decay (Supplementary Figure 2 and Supplementary Note) with a rapid component (19 ± 13 ns) and a slower component (122 ± 13 ns). The decay time in our crystals is significantly longer than in crystals grown from solution methods, indicating fewer traps and higher crystalline quality^30^.

High resolution transmission electron microscopy (HRTEM) was used to further investigate structural uniformity on the atomic and nano scale (Supplementary Note). A cross-sectional TEM sample was prepared parallel to the edge of the cleaved plane using focused ion beam technology as illustrated in Supplementary Figure 3. Single crystallinity and excellent uniformity of CsPbBr_3_ was confirmed using selected area diffraction and HRTEM with FFT. The zone axis of HRTEM in Fig. 1d is determined to be the [010] zone based on the TEM image simulation. No precipitates were found in the as-grown single crystals.

### Asymmetric electrode design

**Fig. 2:**
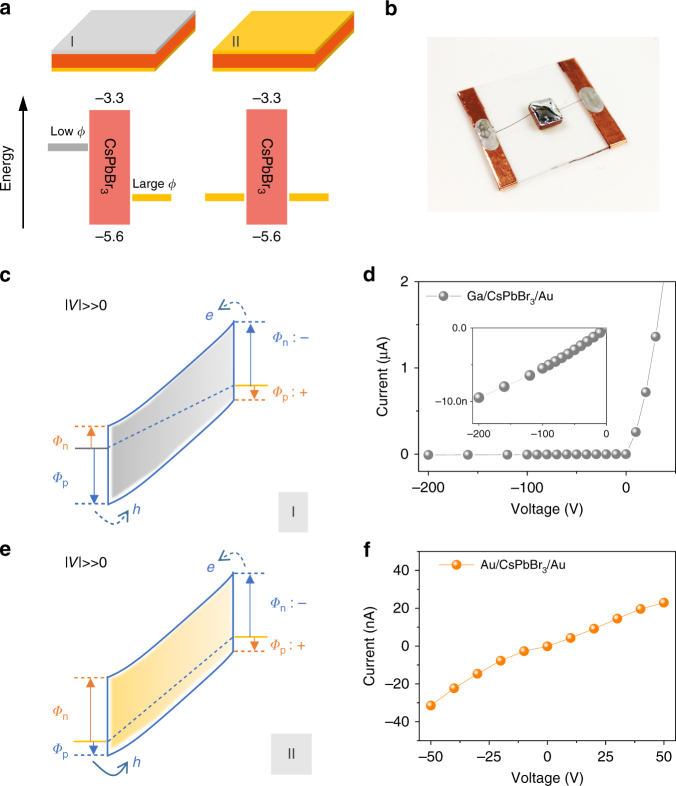
Architecture and electrical properties for CsPbBr_3_ detectors. **a** I and II depict two different designs with asymmetrical and symmetrical electrode materials, and their corresponding energy level diagrams. **b** Typical high performance Ga/CsPbBr_3_/Au detector. **c**, **e** Band diagrams of two different CsPbBr_3_ detectors under large reversed electric fields. Note that the “+,” “−” and *Φ*_n_ and *Φ*_p_ represent the forward and reversed bias and Schottky barrier for electron and hole at the semiconductor–metal interface, respectively. **d**, **f** Typical *I*–V characteristic curve of the Type I (Ga/CsPbBr_3_/Au) and Type II (Au/CsPbBr_3_/Au) detectors

For planar CsPbBr_3_-based hard radiation detectors there are two basic types, Type I and Type II, as illustrated in Fig. 2a. Type I configuration involves an asymmetric choice of detector electrodes where one metal has low work function and the other high, while Type II replies on identical electrodes on both sides of the crystal. Here we demonstrate the efficacy of the Type I structure in fabricating efficient planar radiation detectors. The as-grown CsPbBr_3_ single crystals show *p-*type behavior as judged from the positive Seebeck coefficient, *S*(300 K) = +723(±45) μV K^−1^, at room temperature. To illustrate the energy level diagram in Fig. 2a, we consider that the conduction band minimum and valence band maximum in CsPbBr_3_ are at −3.3 and −5.6 eV, respectively. These energy levels and the *p*-type behavior of the material need to be taken into account in choice of electrodes^31^. Typical high work function electrode metals/materials are Au and Pt but can also be organic or inorganic hole extraction layers (HELs). Low work function metals/materials are usually Al, Ga, and In, but also organic or inorganic electron extraction layers.

Based on the Type I design, we devised an effective electrode configuration for CsPbBr_3_ detectors that can dramatically reduce the dark current by at least three orders of magnitude, while providing impressive temporal stability, especially under high electric field. Ga metal with its relatively small work function (*ϕ*_Ga_ 4.3 eV) was chosen to form a higher Schottky barrier potential *Φ*_p_ under reversed bias. Other materials, such as Al (*ϕ*_Al_ 4.1 eV) and In (*ϕ*_In_ 4.1 eV) and organic HELs, also work similarly without a significant change in energy spectrum. Therefore, for simplicity, we only discuss Ga/CsPbBr_3_/Au detector (Fig. 2b). The assortment of the energy diagram for this metal–semiconductor–metal system generates both higher Schottky barriers *Φ*_n_ and *Φ*_p_ at the semiconductor–metal interface as the device is reversely biased, Fig. 2c. For such a device, the characteristic *I*–*V* curve in Fig. 2d and Supplementary Figure 5a is sublinear and superlinear in the negative and positive voltage range, respectively, owing to deficient injection under reverse bias and excessive injection under forward bias^32^. Even under very large negative bias (−200 V, corresponding to 2000 V cm^−1^), the leakage current is also kept small, around −10 nA (corresponding to 83.3 nA cm^−2^, Fig. 2d). In comparison, as indicated in Supplementary Figure 5c, under higher bias (200 V, 2000 V cm^−1^) the Type II device shows a considerable dark current over 100 μA, corresponding a current density of 1.1 × 10^6^ nA cm^−2^, which is over 10^4^ times higher than that of Type I device. In addition, the dark current of the Type I device is very stable with time, the corresponding *I*–*t* curve is shown in Supplementary Figure 5b. Overall, the greatly suppressed dark current results in lower detector operation noise. Another great feature of the Type I detector is that the Au electrode is serving as the charge collection contact, which can also be advantageous in the fabrication technologies of pixelated or coplanar-grid detectors from the current CdZnTe technology^33^.

The energy diagram for the symmetric Type II electrode configuration under high electric field is depicted in Fig. 2e. In this case, owing to the smaller Schottky barrier *Φ*_p_ at the metal–semiconductor junction holes can be easily injected into the crystal from the positive electrode through tunneling or thermionic emission mechanism^34^ given the *p*-type character of CsPbBr_3_. This can contribute to a large dark leakage current (Fig. 2f). Particularly under high electric field (over 1000 V cm^−1^) for Type II detector the leakage current is usually on the order of microamperes (Supplementary Figure 5c). In addition, the leakage current under high electric field continuously increases over time as extra holes are injected into the system (Supplementary Figure 5d), a temporal instability which prevents the Type II planar detector’s use as a stable and effective γ-ray detector. The resulting background noise from the leakage current of the detector will ruin the energy resolution of the spectrum and the typical γ-ray spectra obtained from the Type II detector will be ill defined at best (Supplementary Figure 6).

### γ-ray spectral performance

Under a 59.5 keV ^241^Am γ-ray source (Supplementary Figure 7a), Type I device (Ga/CsPbBr_3_/Au) showed well-resolved spectroscopic response with the full width at half maximum (FWHM) in percentage of 9.6(±0.5)%. The applied voltage (−150 V, corresponding to a field of 1667 V cm^−1^) and the induced charge *Q* collection were both on the Au cathode, while the γ-ray irradiated Ga anode was grounded. Thus, the measured signal is generated mainly by holes drifting through the entire thickness of the detector. Distinct charge transport properties for electron and hole collection modes are shown in Supplementary Figure 7.

**Fig. 3:**
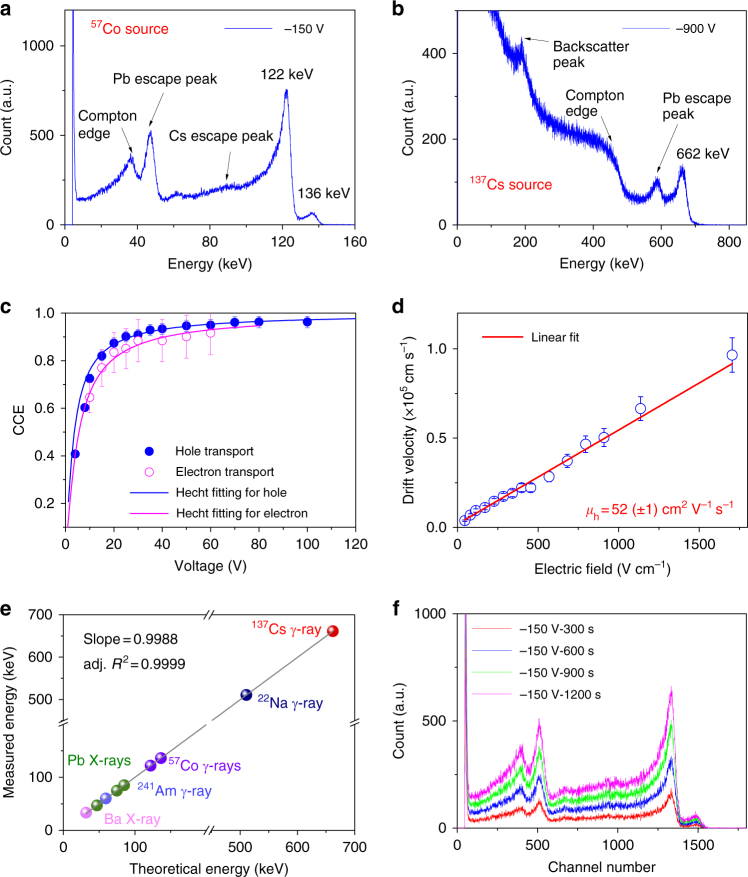
γ-ray response of Type I CsPbBr_3_ detectors to different radioactive isotopes. **a** Energy-resolved spectrum of ^57^Co γ-ray source with a characteristic energy of 122 keV using a shaping time of 2 μs. The dimension of the detector is 3 × 3 × 0.90 mm^3^. **b** Energy-resolved spectrum of ^137^Cs γ-ray source with the characteristic energy of 662 keV with a shaping time of 0.5 μs. The dimension of the detector is 4 × 2 × 1.24 mm^3^. **c** The mobility-lifetime fitting for electron and hole according to the Hecht equation based on the spectra indicated in Supplementary Figure 7. The error bars in CCE arising from obtaining the peak/shoulder channel number represent ±3% and ±10% errors for hole and electron, respectively. **d** Hole mobility *μ*_h_ of CsPbBr_3_ evaluated from the rise time distribution versus applied bias according to the equation *μ* = *d*^2^/(*t*_r_ × *V*), where the *d*, *t*_r_, and *V* are the detector thickness, the rise time, and applied voltage, respectively. The drift velocity is calculated as *d*/*t*_r_. The distribution histogram for corresponding rise time is indicated in Supplementary Figure 12. The error bars in carrier drift velocity represent ±10% errors arising from obtaining rise time. **e** Highly linear detection response of CsPbBr_3_ planar detectors to different radiation sources and the corresponding spectra are shown in Supplementary Figure 10. **f** The stability of spectrum of CsPbBr_3_ detector over a period of 20 min under ^57^Co γ-source measured with a shaping time of 2 μs and applied voltage of −150 V

When exposed to a 0.2 mCi ^57^Co γ-ray source with a characteristic energy of 122 keV, the CsPbBr_3_ detector also delivered a series of excellent ^57^Co γ-ray spectra with extremely low contribution from leakage current noise (Fig. 3a and Supplementary Figure 8) and achieves an energy resolution by FWHM of 4.3(±0.2)% under −150 V. The above results were obtained reproducibly and also confirmed by measurements at Fisk University, where a slightly better energy resolution of 3.9(±0.2)% under −150 V was obtained for the CsPbBr_3_ detector (Supplementary Figure 9). Exposure of our detector to a 5 µCi ^137^Cs source with characteristic energy of 662 keV yielded, as well-resolved spectrum with an energy resolution of 3.8(±0.2)%, see Fig. 3b. The CsPbBr_3_ detector also resolved the spectral line of ^22^Na at 511 keV (Supplementary Figure 10). Such high resolution is in fact comparable to a commercial reference CdZnTe planar detector tested under the same experimental conditions, which demonstrated FWHM of 4.1% for ^57^Co (Supplementary Figure 8) but poorly resolved the 662 keV of ^137^Cs^28^. Indeed, CZT detectors equipped with special contact designs and pulse discrimination, pixelated designs and proper ASIC readout electronics can dramatically improve the energy resolution to a higher level for 662 keV of ^137^Cs to about 1%^35^.

The carrier mobility and mean free drift time (lifetime) product, *µτ*, is extracted from the voltage dependence of the charge collection efficiency (CCE) according to the Hecht equation^36^ from the spectroscopic voltage dependence data for holes and electrons are 1.34 × 10^−3^ and 8.77 × 10^−4^ cm^2^ V^−1^, respectively (Fig. 3c). The *µτ* values for holes and electrons are comparable. This is a unique feature among γ-ray detector materials and is a major advantage for the fabrication of simple detectors. The hole mobility-lifetime product values for our CsPbBr_3_ crystals are one or two orders of magnitude higher than that of the benchmark CdTe and CdZnTe materials, (*µτ*)_h_ approximate 10^−4^–10^−5^ cm^2^ V^−1^, and is comparable to their (*µτ*)_e_ which ranges from 10^−3^ to 1 × 10^−2^ cm^2^ V^−1 13^. We evaluated the hole mobility of our CsPbBr_3_ crystals based on the distribution of rise times *t*_r_ extracted from the transient pulses generated by ^241^Am γ-ray, as has been commonly applied for CdTe and CdZnTe materials^37^. The hole mobility of 52(±1) cm^2^ V^−1^ s^−1^ was obtained which is also consistent with the theoretical polaron hole mobility, 41.3 cm^2^ V^−1^ s^−1^, for CsPbBr_3_^38^.

The experimental mobility-lifetime product, *µτ*, and hole mobility, *µ*, obtained above give an unprecedented long lifetime *τ* for holes of about 25 μs. This strikingly long lifetime is longer than all known radiation detection compound semiconductors. In addition, this is an unprecedented example of a high-spectral resolution γ-ray detecting semiconductor with better hole transport properties than electron transport properties. The presence of strong carrier–phonon coupling in CsPbBr_3_ is a consequence of the dynamic disorder in the perovskite structure^39^, and thus we surmise it contributes to the surprisingly long lifetimes observed.

To determine the spectral linearity of the CsPbBr_3_ detector, we obtained an energy-calibrated spectrum according to the photopeak position of ^57^Co γ-ray (122 keV). A series of photopeaks from different radioactive isotopes are clearly resolved (Supplementary Figure 10) at 32.3 keV (Ba X-ray), 47 keV (Pb escape X-ray), 75 and 85 keV (Pb characteristic X-rays from shielding material used), 59.5 keV (^241^Am γ-ray), 122 and 136 keV (^57^Co γ-ray), 511 keV (^22^Na γ-ray), and 662 keV (^137^Cs γ-ray). As shown in Fig. 3e, the linear (or proportionality) response of such device is excellent, over 0.99. In addition, we also performed an experiment for further demonstration of the simultaneous discrimination of different radioisotopes under identical conditions, as indicated in Supplementary Figure 11. Moreover, the average ionization energy (*ε*_pair_) defined by the average minimum energy required to ionize an electron–hole pair in CsPbBr_3_ is determined to be around 5.3 eV (Supplementary Figure 13).

Our CsPbBr_3_ detector has demonstrated excellent stable response over time. Over a continuous operation period of 1200 s, the peak channel number, count rate (counts per second) and FWHM for ^57^Co γ-ray spectrum were unchanged (Fig. 3f and Supplementary Figure 14), confirming that polarization induced by charge trapping is surprisingly absent for devices with Type I design. The feature of high defect tolerance and low impurity concentration in the CsPbBr_3_ crystals likely play an important role in suppressing polarization under detector operating conditions. The super-long hole lifetime (25 μs) is good evidence for lacking deep-level traps, which will in general substantially reduce the carrier drift lifetime. Thermally stimulated current (TSC) spectroscopy (Supplementary Figure 15 and Supplementary Note) for our CsPbBr_3_ single crystals further confirmed the very low trap density (between 4.11 × 10^14^ cm^−3^ and 5.29 × 10^15^ cm^−3^) and lack of deep-level defects. As indicated in Supplementary Table 2, only five traps with energy level below 0.28 eV were identified.

A relatively long-term detection operation test was also carried out and the CsPbBr_3_ detector demonstrated excellent stability by a continuous operation period over 120 h with the same count rate was retained, while the channel number of the ^57^Co γ-ray photopeak was reduced slightly by 3.4% (Supplementary Figure 16). Detector performance of CsPbBr_3_ detectors fabricated from different batches of crystals grown under the same growth conditions have demonstrated highly resolved spectral peaks under ^57^Co γ-ray irradiation reproducibly (Supplementary Figure 17). The robustness and reproducibility of the crystal growth and device design could definitely push the CsPbBr_3_ to the leading room temperature radiation detection material considering the promise of low cost, easy scale-up and high performance (Supplementary Table 3).

## Discussion

In conclusion, we demonstrated the high resolution γ-ray detection response from high-quality perovskite CsPbBr_3_ perovskite single crystals. We obtained crack free, large single crystal ingots with very high purity (below 10 p.p.m. of total impurities) and low defect density using an optimized scaled-up growth method. Deploying a special device design with asymmetrical electrode materials effectively suppresses the dark leakage current under high electric field and enables the CsPbBr_3_ planar detectors to achieve remarkable energy resolving capabilities. Specifically, the CsPbBr_3_ detector resolved the 59.5 keV (^241^Am γ-ray), 122 and 136 keV (^57^Co γ-ray), 511 keV (^22^Na γ-ray), and 662 keV (^137^Cs γ-ray) lines with best spectral resolution of 3.8%. This high performance is achieved by an unprecedentedly long hole lifetime (over 25 μs) and a superior mobility-lifetime product for holes (1.34 × 10^−3^ cm^2^ V^−1^). Furthermore, CsPbBr_3_ detectors demonstrate long-term stability without observable significant polarization. Because of these promising properties (easy scale-up, low cost and high performance), we believe CsPbBr_3_ is an exceptionally viable candidate for next generation room temperature radiation detectors and possibly displace the leading but problematic materials CdZnTe and TlBr.

## Methods

### Materials

Chemicals in this work were used as obtained: Cesium bromide, 99.999%, Sigma-Aldrich; Lead bromide, 99.999%, Sigma-Aldrich.

### Bridgman growth of CsPbBr_3_ from its melt

Polycrystalline CsPbBr_3_ was prepared directly from CsBr and PbBr_2_ by a solid-state reaction with a 1:1 stoichiometric ratio at 580 °C. For a small scale (less than 40 g), the synthesis and growth were done in the same fused silica tube to reduce unintended contamination that might occur in multistep process. The silica tube for Bridgman growth was of 11 mm inner diameter with a conical shaped end. All processes for synthesis and crystal growth were done in vacuum, with an original pressure less than 10^−4^ Torr (at room temperature). The sealed silica tube was placed on a support rod, which moved downwards with a geared translation system inside a two-zone vertical furnace for single crystal growth. The silica tube was heated up to 590 °C in 10 h and held at that temperature for 12 h for overheating to avoid heterogeneous nucleation. A temperature of 15–30 K of melt overheating helps to reduce excess nucleation centers and suppress the formation of structural defects. The temperature profile was also optimized to avoid thermal-induced cracks and possible constitutional supercooling, thereby enhancing the single crystal yield of CsPbBr_3_. Once the silica tube completely passed with a dropping speed of 0.5–2 mm h^−1^ through the temperature gradient region (with a typical temperature gradient of 5–20 K cm^−1^), the ingot was cooled down to 200 °C in 20 h. Owing to the existence of phase transitions, the ingot was then cooled down to room temperature with a cooling rate of 2–5 K h^−1^. It should be noted that excessive temperature gradient and dropping speed and cooling rate likely result in cracks and final fracture of the ingots (Supplementary Figure 4).

### Sample processing

The CsPbBr_3_ crystal ingots were cut into circular wafers perpendicular to the growth direction and to desired shapes of 0.5–2 mm thickness using a Struers Accutom-50 waferizing saw equipped with a 300 µm wide diamond-impregnated blade. The sample surfaces were then polished mechanically with successively fine SiC grinding papers down to 1 µm particle size.

### Electrode preparation

The polished CsPbBr_3_ crystals were rinsed in toluene to remove any possible surface contamination before the electrode deposition. The Au electrode was prepared by either brushing Au paint or thermal evaporation through a shielding mask onto CsPbBr_3_ samples (30–100 nm thickness for evaporation). For the Ga electrode, it was simply prepared by brushing liquid Ga metal on the surface of the crystal. The electrodes were connected by Cu wire to the outer collection circuit.

### Electrical properties and detector performance measurements

The *I*–*V*characteristic curves were measured by a Keithley 6517B electrometer under dark condition, where resistivity was calculated in the low bias range of −1 to 1 V. For detector performance measurements, the γ-ray sources employed were 1 µCi ^241^Am 59.5 keV, 0.2 mCi ^57^Co 122 keV, 50 µCi ^22^Na 511 keV, and 5 μCi ^137^Cs 662 keV γ-rays. eV-550 preamplifier with bias supply ranging from 4 to 1000 V (or −4 to −1000 V) was connected to the detectors placed in a shielding box. Note that voltage was applied to the bottom contact while the top contact was irradiated with γ-rays. The signal processing and generation of the γ-ray spectrum was described in our previous work^40^. The mobility-lifetime product (*μτ*) was calculated according to the Hecht equation^36,40^, $\eta = \frac{Q}{{Q_0}} = \mu \tau \frac{V}{{d^2}}(1 - \mathrm{e}^{ - \frac{{d^2}}{{\mu \tau V}}})$, where *η* is the CCE, *Q* and *Q*_0_ is the maximum and theoretical saturated channel number of photopeak/shoulder, respectively, *V* is applied bias and *d* is the thickness of the detector. The mobility was estimated using pulse height spectroscopy^40^. We used the average rise time based on the distribution of rise times under each bias to estimate the mobility.

### Data availability

The data that support the findings of this study are available from the corresponding author upon reasonable request.

## Electronic supplementary material

Supplementary Information
