# Supplementary material for: High spectral resolution of gamma-rays at room temperature by perovskite CsPbBr3 single crystals
Source: Nat Commun. 2018 Apr 23;9:1609. doi: 10.1038/s41467-018-04073-3 (PMC5913317; doi:10.1038/s41467-018-04073-3)
Supplement: Supplementary file 1 — Supplementary Information [file 41467_2018_4073_MOESM1_ESM.pdf]

Supplementary Information

High Spectral Resolution of Gamma-rays at Room Temperature by Perovskite $CsPbBr_3$ Single Crystals

He *et al.*

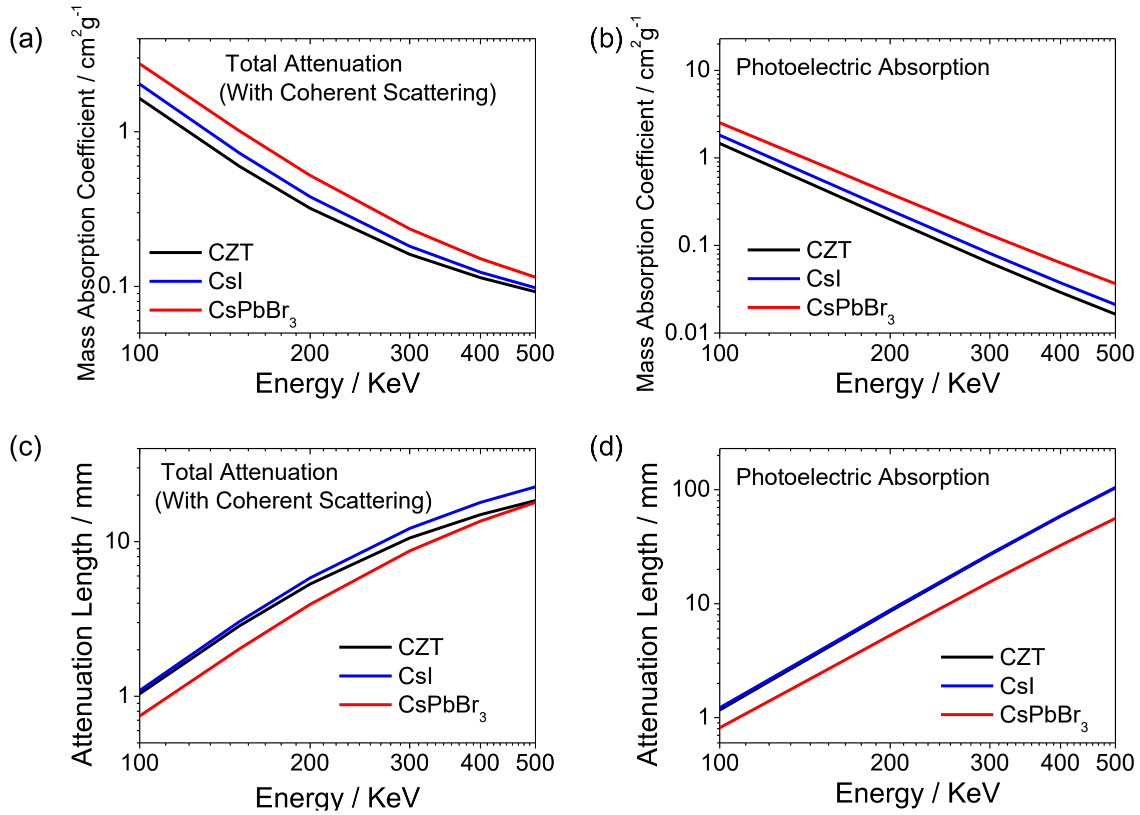

Supplementary Figure 1. Mass absorption coefficient and attenuation length for CsI, CdZnTe and $CsPbBr_3$. [1] For ^{57}Co γ-rays with characteristic energy of 122 keV, the total attenuation lengths are 1.70 mm for CdZnTe, and 1.20 mm for $CsPbBr_3$, respectively.

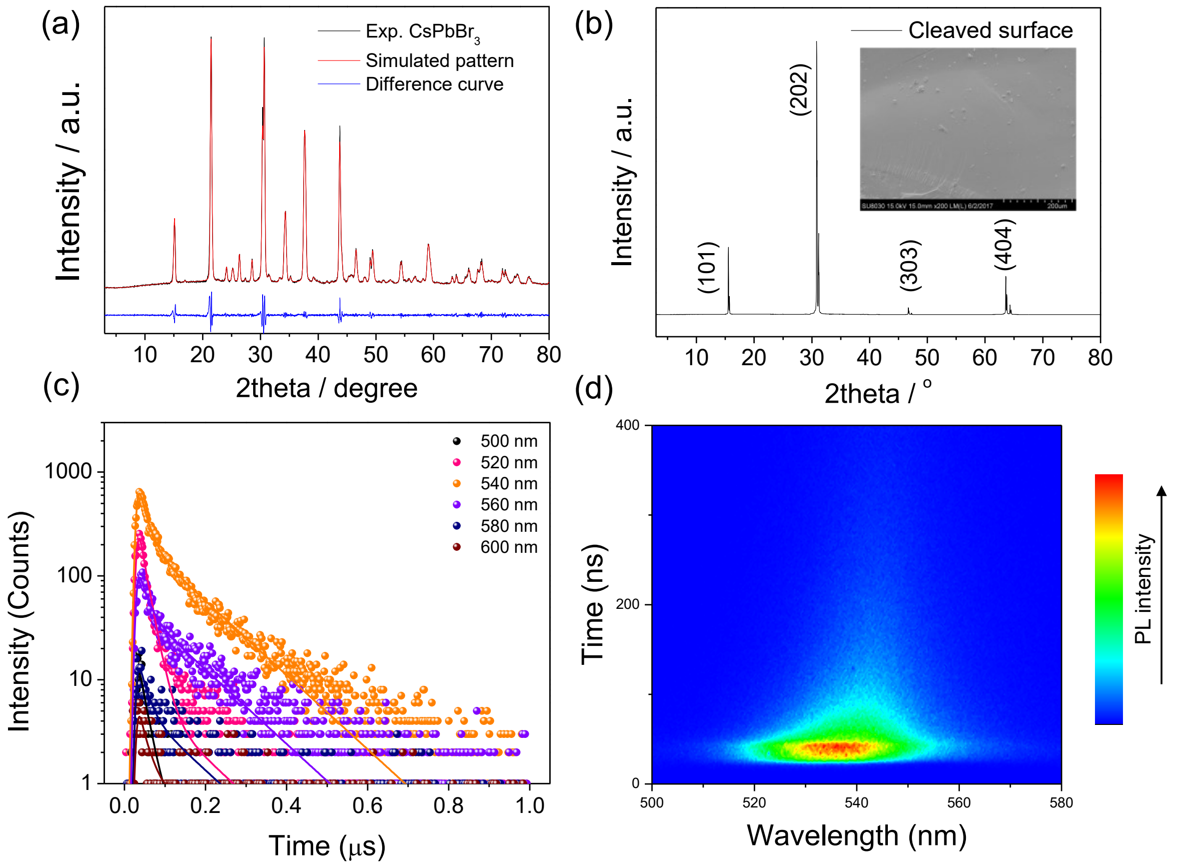

Supplementary Figure 2. (a) Power X ray diffraction patterns (PXRD) of ground $CsPbBr_3$ crystals. The simulated pattern is based on the room temperature orthorhombic phase [2]. The refined lattice parameters from the PXRD pattern herein is: a=8.2420(3) Å b=11.7390(4) Å, and c=8.1934(4) Å, $\alpha=\beta=\gamma$=90°, in good agreement with the literature values of [2] is a=8.2440(6) Å b=11.7351(11) Å c=8.1982(8) Å , $\alpha=\beta=\gamma$=90°. (b) The indices of the basal (*h0h*) reflections observed of a cleaved $CsPbBr_3$ surface using XRD. Inset is the SEM image of the cleaved $CsPbBr_3$ surface. The SEM observation revealed no inclusions were found on the cleaved surface. The dispersed particles are the small $CsPbBr_3$ pieces attached on the surface during cleaving of the crystals. (c) TRPL kinetic experimental data and fits of the $CsPbBr_3$ single crystal collected with a 1 μs experimental window. The symbols depict the experimental kinetic data and the lines depict the kinetic fits. (d) Contour plot depicting the PL decay of the $CsPbBr_3$ single crystal.

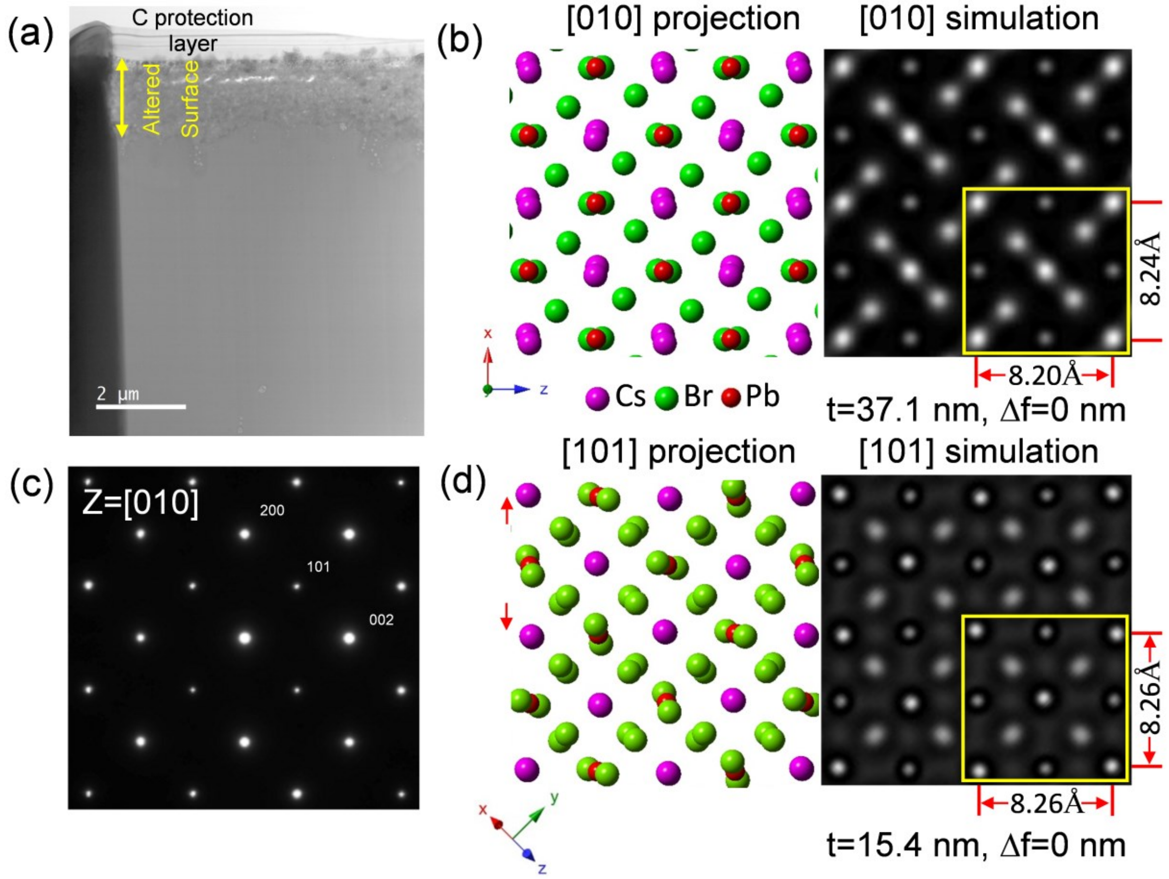

Supplementary Figure 3. (a) Bright field STEM images with SAD in (c) of single crystalline $CsPbBr_3$ grown using Bridgman method. (b) and (d) projection lattice view and TEM image simulation of supercell on [010] and [101] zone respectively. Please note that thickness of [010] and [101] zone supercell slab in (b) and (d) are 37.1 nm and 15.4 nm and simulated at zero defocus. Yellow squares are drawn to compare HRTEM in Figure 1f and image simulations in (b) and (d).

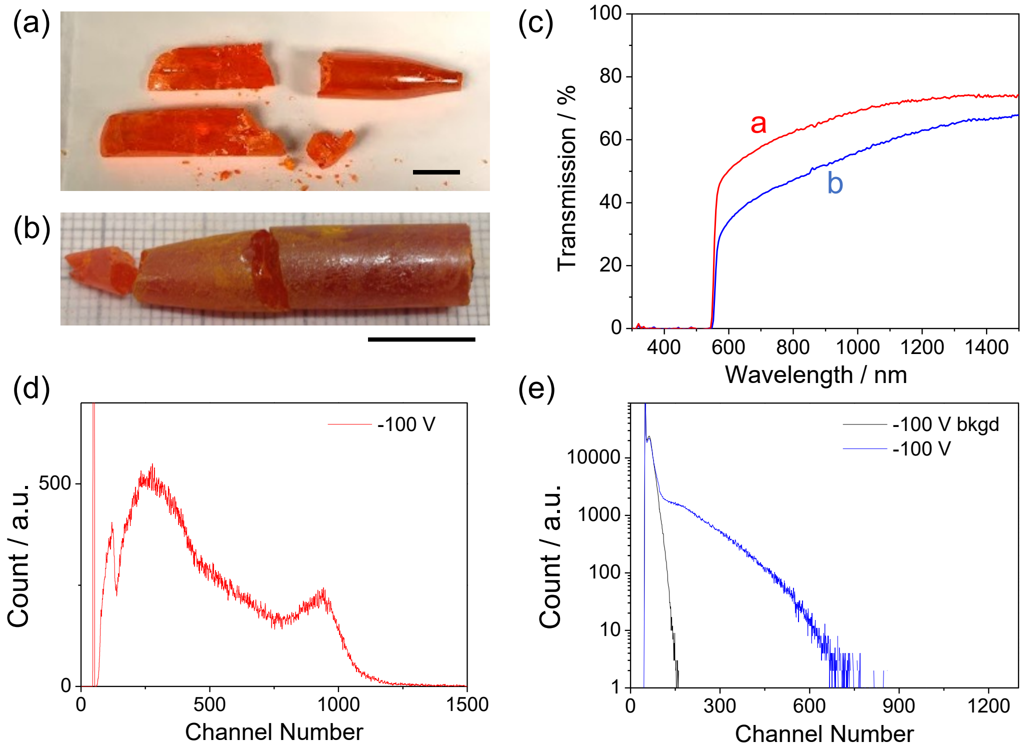

Supplementary Figure 4. (a) $CsPbBr_3$ ingot grown with a dropping speed of about 1 mm h^{-1} with cooling rate of about 50 K h^{-1}. The as-grown ingot contains a long crack parallel to the growth direction causing the ingot to rupture after removing the crystal from the crucible. (b) $CsPbBr_3$ ingot grown with a dropping speed of 3 to 5 mm h^{-1} with cooling rate of over 100 K h^{-1}. The as-grown ingot contains cracks causing the ingot to break into pieces. Owing to the formation of cracks, twins and other possible microstructural defects, the ingots become less transparent with lower optical transmission as shown in (c). The scale bars in (a) and (b) represent 1 cm. (c) Optical transmission spectrum for $CsPbBr_3$ single crystals with thickness of 2 mm obtained in (a) and (b), respectively. Both of the crystals show poorer optical transmission than the crystal shown in Figure 1b, demonstrating the higher quality afforded by the slow dropping rate and cooling rate. (d) and (e) ^{57}Co γ-ray spectra obtained by $CsPbBr_3$ detectors fabricated from the ingot (a) and (b), respectively. The detectors were fabricated with Ga and Au electrodes (Type I). The shaping time used here was 6 μs for both spectra. The crystals grown using slow dropping speed and cooling rate indicated spectroscopic response but poor energy resolution (about 20%) while the crystal obtained with the fast growth was not able to resolve the γ-ray lines at all. As compared to the results in Figure 3, the crystal obtained under the improved growth conditions show superior detector performance, confirming the critical role of the crystal growth process to the final detector properties.

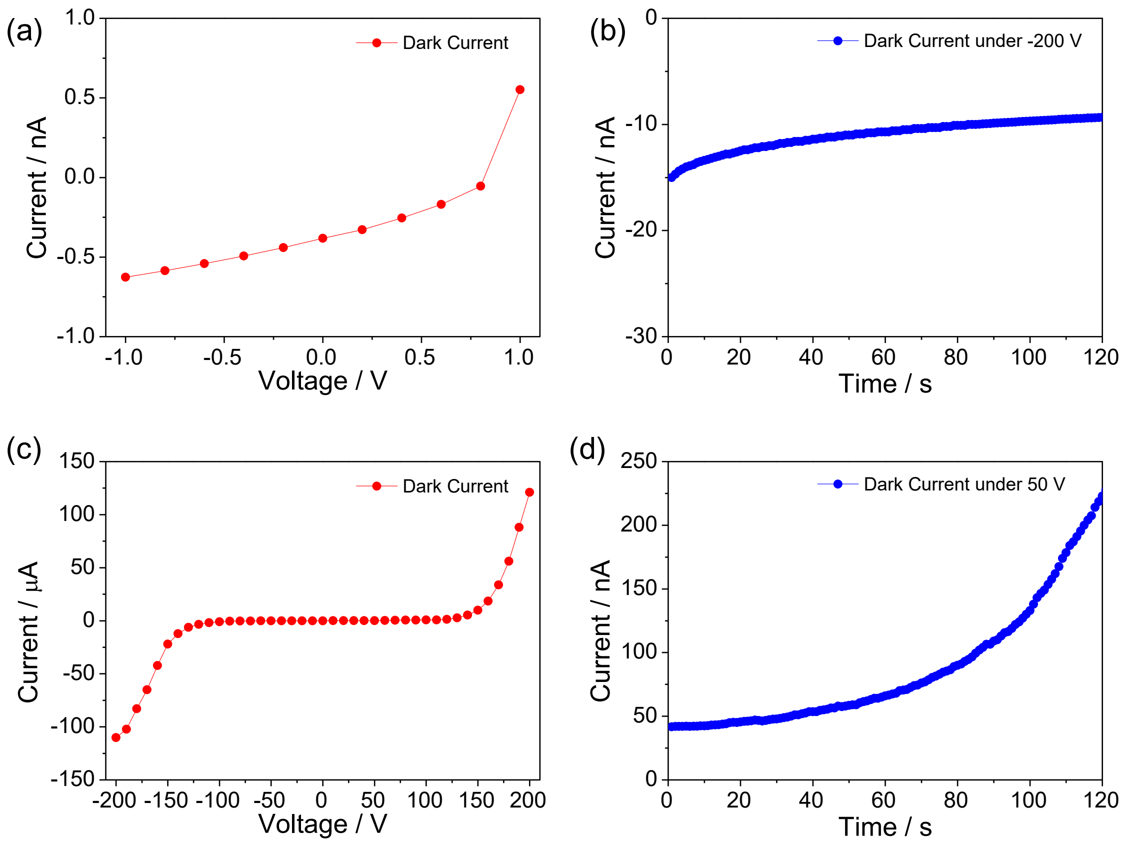

Supplementary Figure 5. (a) *I-V* curve in the small bias range of Type I detector shown in Figure 2d. (b) Dark current variation with time under negative 200 V. The dimension of Type I detector is 4×3×1 mm^3 with fully covered electrodes on both parallel surfaces. (c) *I-V* curve in the large bias range of Type II detector shown in Figure 2f. (d) Dark current variation with time under positive 50 V. The dimensions of Type II detector is 3×3×1 mm^3 with fully covered electrodes on both parallel surfaces.

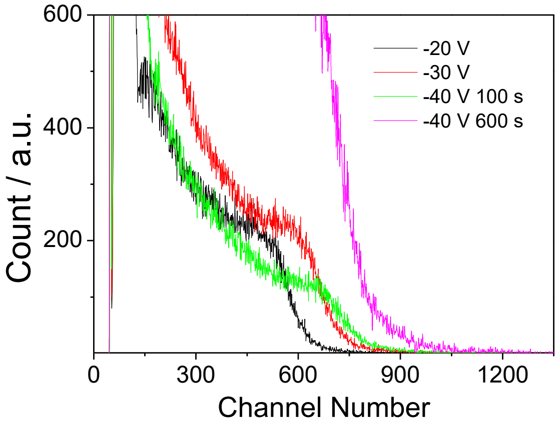

Supplementary Figure 6. ^{57}Co γ-ray spectrum obtained by $CsPbBr_3$ detectors by using Au electrodes on both sides (Type II). The dimension of the sample is 6×6×2.86 mm^3. The shaping time used was 6 μs. The applied negative bias was on the bottom electrode, so the induced charges were mainly contributed from hole transport. Because of the relatively low resistivity, the highest bias applied is -40 V and the detector cannot produce a stable signal for a time period over 100 s.

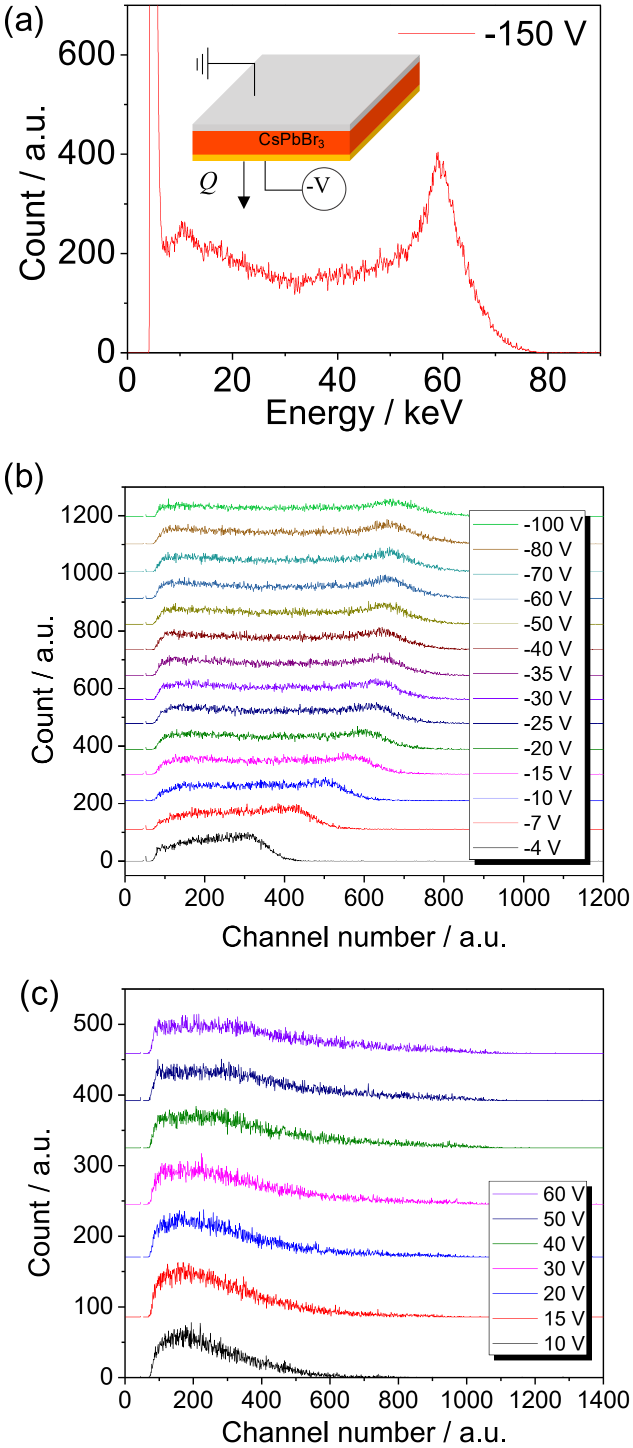

Supplementary Figure 7. (a) Energy-resolved spectrum of ^{241}Am γ-ray source with a characteristic energy of 59.5 keV using a shaping time of 2 μs. The inset is the schematic principle of the detection measurement. The energy spectra of 59.5 keV ^{241}Am γ-ray source from $CsPbBr_3$ detector with a shaping time of 6 μs under (b) negative bias (hole collection) and (c) positive bias (electron collection). While increasing the applied bias, the spectra shifted to the higher channel number, indicating the improvement of charge collection efficiency (CCE).

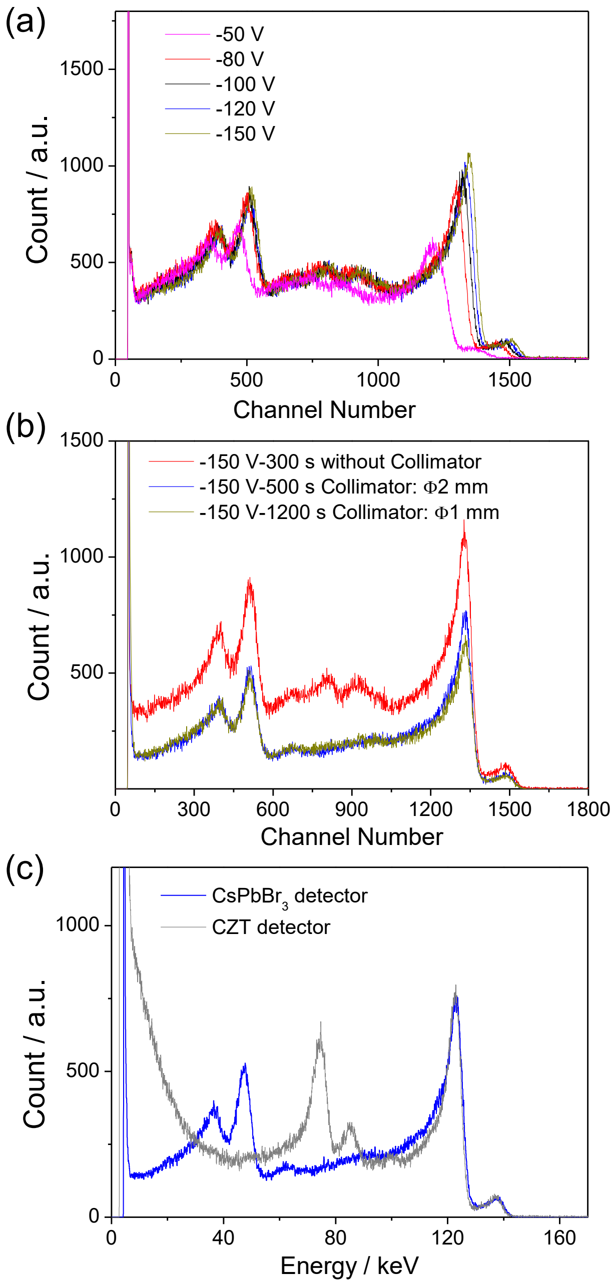

Supplementary Figure 8. (a) ^{57}Co γ-ray spectra of $CsPbBr_3$ detector obtained under various biases with a shaping time of 2 μs. (b) The ^{57}Co energy spectra influenced by the different collimation conditions with a shaping time of 2 μs. The collimation suppresses the spectra background and eliminates the Pb escape X-ray fluorescence from the lead shielding material. The collimation also reduces the counting rate owing to the loss of γ-ray intensity and the '300 s, 500 s and 1200 s' represent the spectrum collection time. Note that spectra in Figure 3a and 3f were measured by using a lead collimator with size of 2 mm diameter. (c) Comparison between the $CsPbBr_3$ detector biased at -150 V and commercial CdZnTe detector biased at 300 V with dimensions of 5×5×2 mm^3 from eV products (now named Kromek [3]) tested under the same system configurations with a shaping time of 2 μs. Note that the 122 keV peak position was normalized for better clarification.

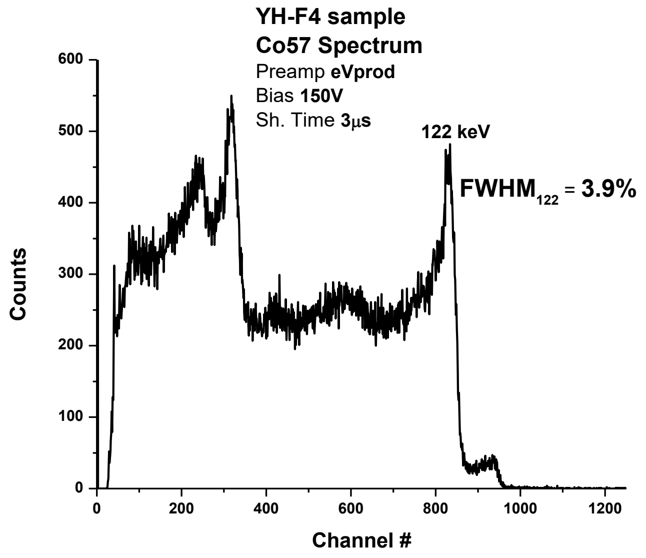

Supplementary Figure 9. ^{57}Co spectrum measured by $CsPbBr_3$ detector at Fisk University. The energy resolution obtained here is 3.9 (±0.2) %, corresponding well with our results obtained at NU. Note that the $CsPbBr_3$ detector was made at NU and then delivered to Fisk University for further measurement.

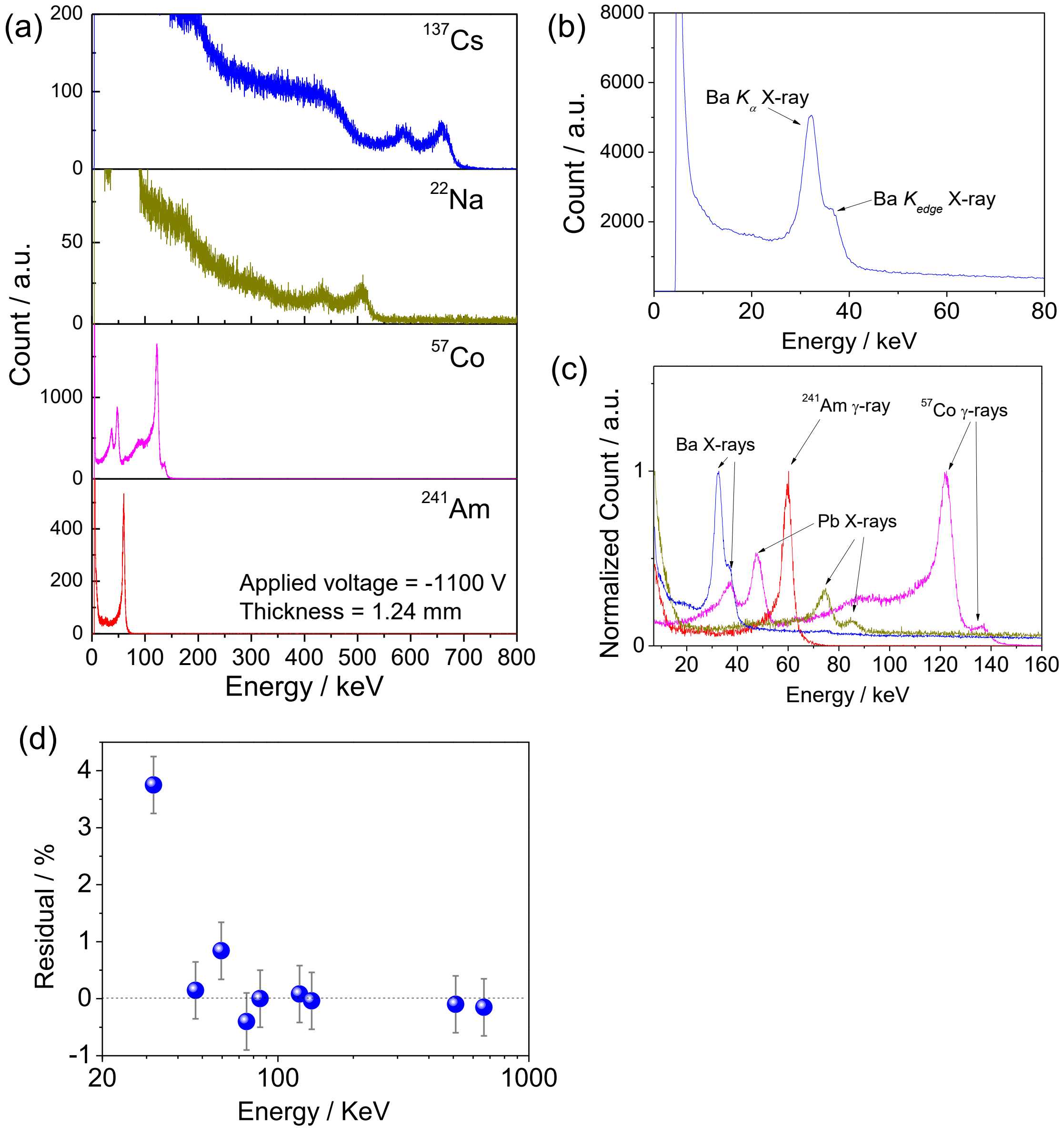

Supplementary Figure 10. (a) Resolved γ-ray spectra from four different sources. The spectra were energy-calibrated according to the photopeak position of ^{57}Co γ-ray (122 keV). The detector used here is the same device that is used in Figure 3b. Each spectrum was collected under the exact same test conditions (-1100 V, 0.5 μs shaping time). The γ-ray sources employed were 1 μCi ^{241}Am 59.5 keV and 0.2 mCi ^{57}Co 122 keV and 5 μCi ^{137}Cs 662 keV γ-ray sources. (b) Ba K*a* and K-edge X-ray (32.2 and 37.4 keV, respectively) from the ^{137}Cs 662 keV decays. (c) Comparison of spectral lines including escape peaks and decay peaks (e.g. Ba X-ray from ^{137}Cs decay and Pb escape X-ray excited by the ^{57}Co γ-ray) from which the excellent linearity of the $CsPbBr_3$ planar detector can be obtained. (d) The linearity response of $CsPbBr_3$ detector revealed by the residuals, i.e., the percentage deviation from the linear fit in Figure 3e. The residual in percentage is defined as ((measured energy-theoretical energy) / theoretical energy × 100%).

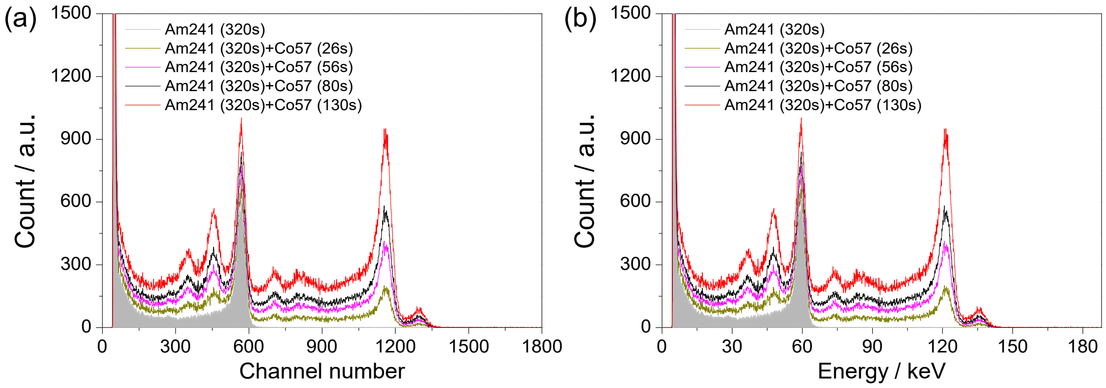

Supplementary Figure 11. (a) Operation of Type I $CsPbBr_3$ detector under dual γ-ray sources simultaneously (1 μCi ^{241}Am 59.5 keV and 0.2 mCi ^{57}Co 122 keV γ-ray sources). The spectra here are the raw data without any correction. The present source for the first 320 s was ^{241}Am 59.5 keV γ-ray source shown in the gray shaded region. Then the ^{57}Co 122 keV γ-ray source was also added to the detection window and it was found that the detector resolved all of the characteristic peaks at the same time. Note that the higher activity of the ^{57}Co source is responsible for the disparity in counting rate. (b) Calibrated spectra of $CsPbBr_3$ detector under dual γ-ray sources according to ^{241}Am 59.5 keV γ-ray photopeak position. It is a simple but effective demonstration of the identification of radioisotopes using $CsPbBr_3$ detector using given bias conditions and shaping time.

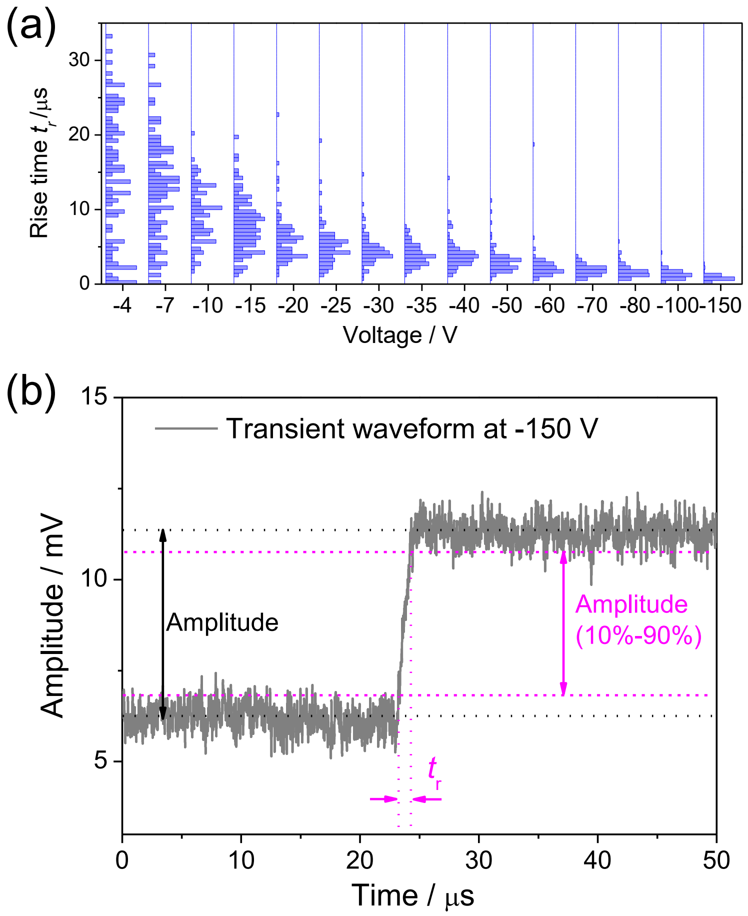

Supplementary Figure 12. (a) Rise time distribution versus applied bias under ^{241}Am γ ray source of $CsPbBr_3$ detector, based on 100 transient waveforms under each bias. The thickness of the sample is 0.90 mm. (b) A typical transient waveform from preamplifier for extracting the rise time under -150 V, the corresponding t_r here is 0.82 μs.

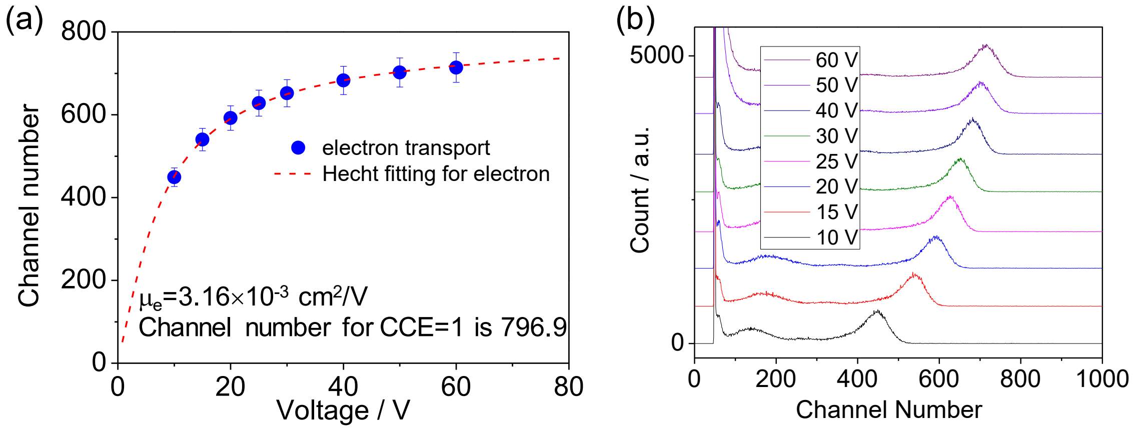

Supplementary Figure 13. Charge collection efficiency fitting (a) according to Hecht equation and the corresponding ^{241}Am 59.5 keV spectrum (b) measured by a CdZnTe detector from eV products company. The measurement condition is exactly the same to the spectrum obtain in Supplementary Figure 7b. The $\mu\tau$ for electron of CdZnTe detector is estimated to be 3.16×10^{-3} cm^2 V^{-1}. The estimated saturated channel number for CCE=1 is 796.9. According to Supplementary Figure 7b, the saturated channel number for CCE=1 of $CsPbBr_3$ detector is 686.8. The $\varepsilon_{\text{pair}}$ for CdZnTe used for calculation is 4.6 eV[4], so the $\varepsilon_{\text{pair}}$ for $CsPbBr_3$ determined here is 5.3 eV, according to the method described in reference [5]. This was done by comparing the peak position of 59.5 keV γ-ray recorded with a CdZnTe detector used as a standard whose ε_{pair} is well known. [6] The ε_{pair} in 5.3 eV for $CsPbBr_3$ is smaller than the one predicted by the Klein's semi-empirical relationship ($\varepsilon_{pair} \approx 3E_g$, 6.84 eV), and is similar in behavior to other halide semiconductors, such as HgI_2, TlBr and PbI_2. [7]

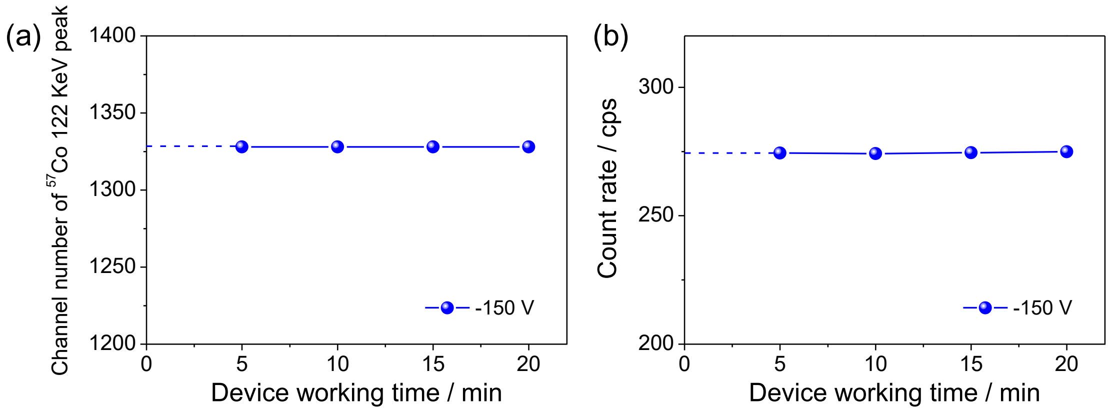

Supplementary Figure 14. The stability of the detector operation with time continuously. (a) peak channel number and (b) count rate (counts per second).

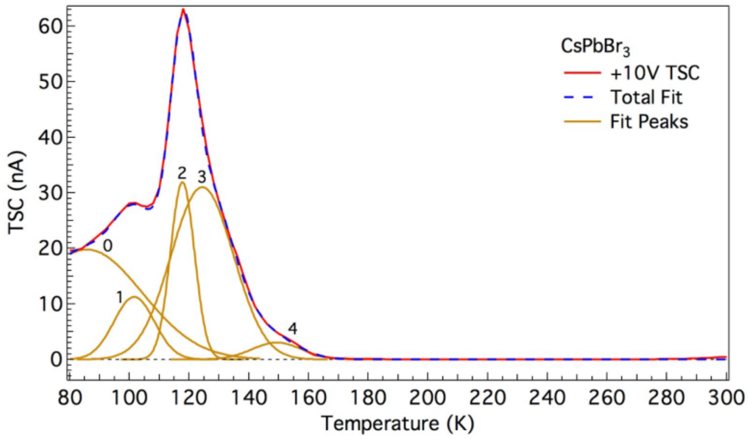

Supplementary Figure 15. Thermally stimulated current at +10 V in $CsPbBr_3$ after excitation by a 405 nm laser, with a five-peak fit.

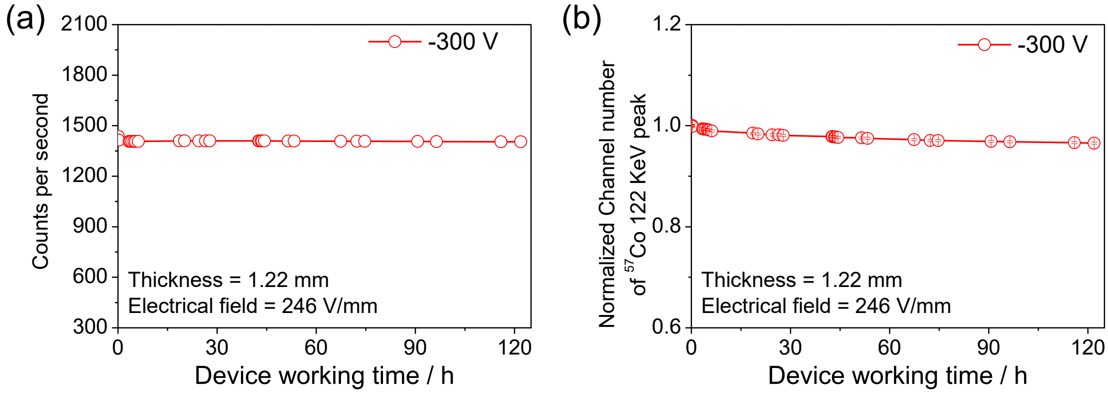

Supplementary Figure 16. The stability of the Type I detector operation over a period of over 120 h continuously. (a) count rate (counts per second) and (b) the normalized peak channel number of the ^{57}Co 122 keV γ ray.

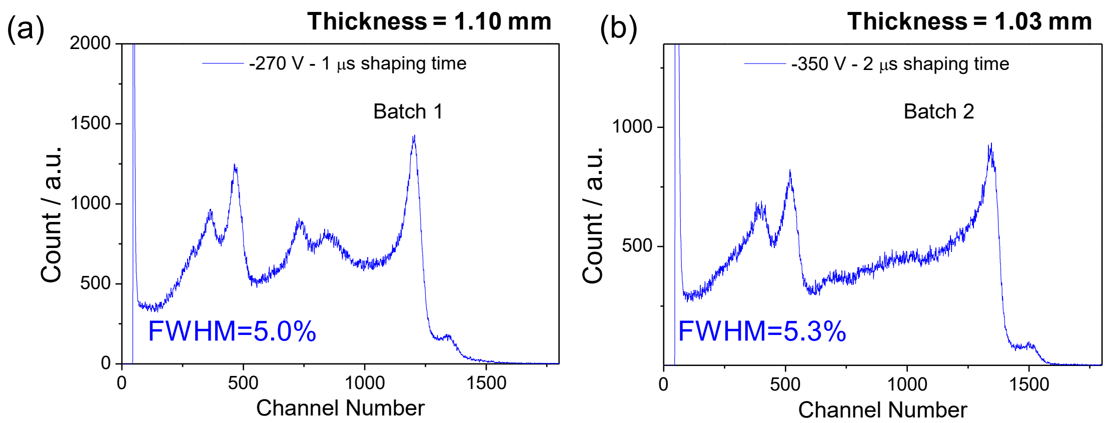

Supplementary Figure 17. Detector performance of $CsPbBr_3$ detectors fabricated from different batches (a-b) of crystals grown under the same growth conditions. Well-resolved spectral peaks under ^{57}Co γ-ray irradiation were reproducibly obtained from different batches of $CsPbBr_3$ crystals. The mobility-lifetime product for hole carriers varied between 6×10^{-4} cm^2 V^{-1} and 1.4×10^{-3} cm^2 V^{-1} among different batches.

Supplementary Table 1. Element analysis performed by Glow Discharge Mass Spectrometry (GDMS) for 69 elements in $CsPbBr_3$ single crystals. This method is an extremely sensitive technique to determine low level trace impurities. GDMS results tabulated with a 'less than' sign are detection limits. For most of the elements, their concentration lies below the detection limits. The total impurity level determined, excluding the elements outside the detection limit, is below 10 ppm *wt.* (μg g^{-1}).

Elements	Concentration / ppm wt.	Elements	Concentration / ppm wt.	Elements	Concentration / ppm wt.
Li	0.05	Ag	0.26	As	< 0.1
Be	< 0.005	Cd	< 0.1	Se	< 0.1
B	< 0.005	In	Binder	Br	Matrix
F	< 1	Sn	< 0.5	Rb	1.3
Na	0.36	Sb	< 0.5	Sr	0.03
Mg	0.03	Te	< 0.1	Y	0.02
Al	0.04	I	< 0.8	Zr	< 0.005
Si	0.03	Cs	Matrix	Nb	0.01
P	< 0.01	Ba	< 0.05	Mo	< 0.005
S	0.06	La	< 0.05	Ru	< 0.005
Cl	6.8	Ce	< 0.05	Rh	< 0.01
K	< 0.5	Pr	< 0.05	Pd	< 0.01
Ca	0.72	Nd	< 0.05	W	< 0.05
Sc	< 0.005	Sm	< 0.05	Re	< 0.01
Ti	0.02	Eu	< 0.5	Os	< 0.01
V	< 0.005	Gd	< 0.05	Ir	< 0.01
Cr	< 0.005	Tb	< 0.05	Pt	< 0.5
Mn	< 0.005	Dy	< 0.05	Au	< 0.5
Fe	< 0.005	Ho	< 0.05	Hg	< 0.05
Co	< 0.005	Er	< 0.05	Tl	< 0.1
Ni	< 0.005	Tm	< 0.05	Pb	Matrix
Cu	0.08	Yb	< 0.05	Bi	< 0.5
Zn	< 0.005	Lu	< 0.05	Th	< 0.01
Ga	0.04	Hf	< 0.005	U	< 0.01
Ge	< 0.05	Ta	Source		

Supplementary Table 2. TSC peak temperatures and calculated trap energies and densities for $CsPbBr_3$ single crystal.

	T_m (K)	E_t (eV)	N_t ($\times 10^{15}$ cm^{-3})
peak 0	86	–	–
peak 1	102	0.18	1.25
peak 2	118	0.21	2.10
peak 3	124	0.23	5.29
peak 4	149	0.28	0.41

Supplementary Table 3. Fundamental properties comparison between the current semiconductors and $CsPbBr_3$ in the field of room temperature radiation detection.

**Materials**	**Density ($g\ cm^{-3}$)**	**E_g (eV)**	**μ_e ($cm^2\ V^{-1}\ s^{-1}$)**	**μ_h ($cm^2\ V^{-1}\ s^{-1}$)**	**$(\mu\tau)_e$ ($cm^2\ V^{-1}$)**	**$(\mu\tau)_h$ ($cm^2\ V^{-1}$)**	**ρ (Ω·cm)**	**HV**	**T_m (°C)**	**Start from**
CdTe[4,8,9]	5.85	1.44	1100	100	3×10^{-3}	2.6×10^{-4}	10^9	50	1092	1970s
CdZnTe [4,8,9]	5.78	1.57	1000	100	7×10^{-3}	9×10^{-5}	10^{10}	60-80	~1100	1990s
TlBr[4,10]	7.56	2.68	30	4	4.1×10^{-3}	4×10^{-4}	10^{11}	<10	460	1980s
$CsPbBr_3$	4.85	2.28	N/A	52	$>8\times10^{-4}$	$>1\times10^{-3}$	10^9	25	550	2013

Supplementary Note

Steady-State Photoluminescence. Steady-state PL spectra were measured using a HORIBA Nanolog fluorimeter. The sample was excited at 440 nm.

Time-Resolved Photoluminescence (TRPL) spectroscopy. A streak camera system (Hamamatsu C4334 Streakscope) was used to collect the time-resolved photoluminescence (TRPL) data. The instrument response function (IRF) of the experiment is about 201 ps. 440 nm laser pulses were generated by a high repetition rate ultrafast laser system. A one box ultrafast amplifier (Spirit, Spectra-Physics) produces a 1040 nm (100 kHz, 350 fs) fundamental beam that is used to pump a non-collinear optical parametrical amplifier (Spirit-NOPA, Spectra-Physics); this delivers tunable high repetition rate pulses. Frequency doubling of 880 nm by the built-in second harmonic generation module in Spirit-NOPA generated the 440 nm laser pulses used in this experiment. The sample was excited with 440 nm, 0.1 nJ pulses.

The TRPL data are fit using a multiple-wavelength global fitting method in Supplementary Figure 2c. The kinetic data from 500 to 600 nm (in 10 nm intervals) are fit to an A→B→ground state (GS) model. Each wavelength is given an initial amplitude that is representative of the spectral intensity at time t_0, and varied independently to fit the raw data. The time constants and t_0 are shared between the various kinetic data and are varied globally across the kinetic data in order to fit the proposed model. A MATLAB program numerically solves the differential equations through matrix methods[11] and then convolutes the solutions with a measured Gaussian instrument response function with width 0.01267 (FWHM) before employing a least-squares fitting using a Levenberg-Marquardt method to find the parameters, which results in matches to the kinetic data. The fit parameters are then fed into the differential equations, which are solved for the population of the states in the model. The species associated spectra are then generated by deconvoluting the raw data matrix with the populations as functions of time.

Transmission electron microscopy characterization. A cross-sectional TEM sample is prepared using FEI Helios NanoLab™ 600 DualBeam (FIB/SEM). HRTEM and STEM images were obtained using JEOL ARM 200cF equipped with probe Cs-corrector. TEM image simulation of $CsPbBr_3$ on [010] and [101] zones were conducted by QSTEM. [12] Amorphous carbon film for protection layer was deposited using 5kV electron-beam in focused ion beam (FIB), causing surface damage of 1 to 2 µm as can be seen in Bright-Field STEM (Supplementary Figure 3a). In order to interpret zone axis of HRTEM in Figure 1f, $CsPbBr_3$ supercell slabs on [010] and [101] zones were generated with different thickness of 37.1 nm and 15.4 nm respectively, along the zones for TEM image simulation which visualizes projected atomic potential using QSTEM software [12] in Supplementary Figure 3b and 3d. Asymmetries and contrast of 4 Br atom columns surrounding a central Cs atom column within a square array in Figure 1f identical to the [010] zone image simulation in Supplementary Figure 3b unlike the symmetric arrangement of Br atom columns on the [101] zone in Supplementary Figure 3d, which agrees well with the diffraction pattern in Supplementary Figure 3c.

Thermally stimulated current (TSC) measurements. The sample was mounted in a cryostat and cooled under dark conditions to 80 K. Dark current was measured as a function of temperature from 80 to 300 K under +10 V bias with a heating rate of 8 K min^{-1} to get a baseline. Subsequently, the sample was cooled to 80 K and illuminated with a 405 nm laser at 5 mW for 15 minutes to fill any traps that may be present in the sample. The current was then measured from 80 K to 300 K with an identical heating rate of 8 K min^{-1}, and the baseline subtracted to obtain the thermally stimulated current.

Thermally stimulated current spectroscopy was carried out to investigate the nature of defects that may be involved in the charge transport of $CsPbBr_3$. These measurements, carried out from 80 K to 300 K, showed several peaks in the low-temperature region (Supplementary Figure 15). The first peak is used to fit the edge of the spectrum, and is excluded from the analysis because its true maxima may lie below liquid nitrogen temperatures. Nevertheless, the absence of high-temperature peaks indicates that the defect level energies involved are relatively low. The maxima of each peak (T_m) can be used to estimate the trap energy E_t, which is given by Bube as:[13]

$$E_t = kT_m \ \ln\left(\frac{NSv_{th}kT_m^2}{\beta E_t}\right), \qquad (1)$$

where k is the Boltzmann constant, T_m is the temperature of the TSC peak, N is the density of states, S is the capture cross section, v_{th} is the carrier thermal velocity, and β is the heating rate. Following the approach of Fang *et al.*, when $T_m \gtrsim 100$ K and re-trapping can be neglected, the temperature dependence of $N \sim T^{3/2}$ and $v_{th} \sim T^{1/2}$ can be used to simplify Supplementary Equation 1 to the following expression:

$$E_t = kT_m \ln\left(\frac{T_m^4}{\beta}\right). \qquad (2)$$

Using Supplementary Equation 2, the trap energies estimated here are shallow, ranging from 0.18 eV to 0.23 eV, with a relatively weak trap at 0.28 eV (Supplementary Table 2). These are in agreement with recent deep-level transient spectroscopy studies on the related compound $CH_3NH_3PbBr_3$ which found two shallow traps of energy 0.17 eV and 0.20 eV.[14] The carrier concentration of each trap N_t can also be estimated from the total charge collected in the TSC measurement Q_t divided by the effective carrier collection region in the crystal. This region is a measure of the amount of charge that can be expected to escape the trap and reach the electrodes, and thus depends directly on the mobility μ, the carrier lifetime τ, the applied electric field E, and the electrode area A, yielding the relation:[15]

$$N_t = \frac{Q_t}{2\mu\tau qAE}, \qquad (3)$$

where q is the elementary charge. The total charge collected is the time integral of the current peak, defined $Q_t = \int I_{TSC}dt = \frac{1}{\beta}\int_{T_0}^{T} I_{TSC}dT$. Using Supplementary Equation 3 we obtain trap concentrations between 4.11×10^{14} cm^{-3} and 5.29×10^{15} cm^{-3}, indicating a high crystal quality. For reference, high-quality CZT crystals have trap densities in the range 10^{13}–10^{15} cm^{-3}.[16,17] The absence of deeper mid-gap traps and the relatively low defect concentrations of the shallower levels support the detector-grade quality of the crystals, corroborating the excellent response to ^{57}Co γ- ray radiation observed in this work.

Supplementary References

1. https://physics.nist.gov/PhysRefData/Xcom/html/xcom1.html
2. Stoumpos, C. C. *et al.* Crystal Growth of the Perovskite Semiconductor $CsPbBr_3$: A New Material for High-Energy Radiation Detection. *Cryst. Growth Des.* **13**, 2722-2727, (2013).
3. http://www.kromek.com/.
4. Owens, A. Semiconductor materials and radiation detection. *J. Synchrotron Radiat.* **13**, 143-150, (2006).
5. Hitomi, K., Onodera, T., Kim, S. Y., Shoji, T. & Ishii, K. Experimental Determination of the Ionization Energy in TlBr. *IEEE Trans. Nucl. Sci.* **62**, 1251-1254, (2015).
6. Klein, C. A. Bandgap Dependence and Related Features of Radiation Ionization Energies in Semiconductors. *J. Appl. Phys.* **39**, 2029-2038, (1968).
7. Shah, K. S., Lund, J. C., Olschner, F., Moy, L. & Squillante, M. R. Thallium bromide radiation detectors. *IEEE Trans. Nucl. Sci.* **36**, 199-202, (1989).
8. Capper, P., *Properties of Narrow Gap Cadmium-based Compounds*. 1994.
9. Triboulet, R. & Siffert, P. *CdTe and Related Compounds; Physics, Defects, Hetero- and Nano-structures, Crystal Growth, Surfaces and Applications*. 550 (ELSEVIER, 2009).
10. Hitomi, K., Shoji, T. & Ishii, K. Advances in TlBr detector development. *J. Cryst. Growth* **379**, 93-98, (2013).
11. Berberan-Santos, M. N. & Martinho, J. M. G. The integration of kinetic rate equations by matrix methods. *J. Chem. Educ.* **67**, 375-379, (1990).
12. Koch, C. *Determination of Core Structure Periodicity and Point Defect Density Along Dislocations*. (Arizona State University, 2002).
13. Bube, R. H., *Photoconductivity of solids*. Wiley: New York, 1960.
14. Rosenberg, J. W., Legodi, M. J., Rakita, Y., Cahen, D. & Diale, M. Laplace current deep level transient spectroscopy measurements of defect states in methylammonium lead bromide single crystals. *J. Appl. Phys.* **122**, 145701, (2017).
15. Nan, R. *et al.* Investigation on defect levels in CdZnTe : Al using thermally stimulated current spectroscopy. *J. Phys. D: Appl. Phys.* **43**, 345104, (2010).
16. Gul, R. *et al.* Point Defects in CdZnTe Crystals Grown by Different Techniques. *J. Electron. Mater.* **40**, 274-279, (2011).
17. Pak, R. O., Nguyen, K. V., Oner, C., Mannan, M. A. & Mandal, K. C. in *SPIE Optical Engineering + Applications.* 8 (SPIE).
